# Recurrent Ectopic Pregnancies Following Bilateral Partial Salpingectomy: A Case Report

**DOI:** 10.1155/crog/6044663

**Published:** 2026-04-07

**Authors:** Monica Hill, Panagiotis Cherouveim, Shagun Tuli, Jay Holmes

**Affiliations:** ^1^ College of Human Medicine, Michigan State University, East Lansing, Michigan, USA, msu.edu; ^2^ Department of Obstetrics and Gynecology, Hurley Medical Center, Flint, Michigan, USA, hurleymc.com

**Keywords:** ectopic pregnancy, partial salpingectomy, recurrent, residual tubal segments, young age

## Abstract

**Background:**

Ectopic pregnancies can be a life‐threatening condition in early pregnancy. Risk factors include pelvic inflammatory disease, prior ectopic pregnancy, pelvic surgery, and anatomical variations. Although pregnancy after tubal sterilization is uncommon, recurrent ectopic pregnancy after bilateral partial salpingectomy is rare.

**Case:**

A 28‐year‐old G5P1031 African‐American patient who underwent cesarean delivery with bilateral partial salpingectomy. Ten months later, she presented with a right ectopic pregnancy requiring laparoscopic salpingectomy. This was followed by a ruptured left ectopic pregnancy treated with salpingo‐oophorectomy 7 months later.

**Conclusion:**

This case highlights the rare but serious risk of recurrent ectopic pregnancy following partial salpingectomy. Residual tubal segments and young age may contribute to this. Clinicians should consider total salpingectomy when feasible to reduce recurrence and improve long‐term outcomes.

## 1. Introduction

Ectopic pregnancy affects 1%–2% of all pregnancies worldwide [1]. Ruptured ectopic pregnancy is the leading cause of first‐trimester maternal mortality, accounting for 5%–10% of all pregnancy‐related deaths [2]. The fallopian tubes are the most common site of ectopic pregnancy implantation (95%) [2]. Risk factors include pelvic inflammatory disease, previous ectopic pregnancy, previous pelvic surgery, use of assisted reproductive technologies (i.e., IVF), endometriosis, intrauterine device (IUD) use, and anatomical variations [2]. Management for ectopic pregnancy is either medical or surgical, depending on patient stability, imaging, and laboratory findings, and rupture risk [2]. Ruptured ectopic pregnancy requires emergency surgical intervention.

Tubal sterilization is the most common method of permanent contraception used worldwide [3]. Sterilization techniques include complete salpingectomy, partial salpingectomy, and tubal ligation. Complete salpingectomy excises the entire fallopian tube [4]. Partial salpingectomy excises the mid portion of the fallopian tube and is commonly performed using the Parkland or Pomeroy methods [4]. Tubal ligation redirects or occludes the divided ends of the fallopian tube, as seen in the Uchida or Irving methods [4]. The Parkland and Pomeroy methods are the preferred methods for sterilization due to their simplicity and shorter operative times [4, 5]. Older sterilization methods, such as Medlener and Kroenern, are no longer recommended due to their high failure rates [5]. The benefits of sterilization are high contraceptive efficacy, disease prevention, and reduced risk of ovarian cancer [6]. Risks vary by surgical approach and include unintended pregnancy, ectopic pregnancy, infection, injury to surrounding structures (i.e., bowel, bladder, and major vessels), and regret [6].

Clark et al. [4] reported a 10‐year overall failure rate of 7.5 per 1000 procedures for the Parkland and Pomeroy methods. However, when looking at the 10‐ and 15‐year postprocedure ectopic pregnancy rates across all methods, the rates were 2.4 and 2.9 per 1000, respectively [7]. When accounting for patient demographics, a 10‐year cumulative probability of ectopic pregnancy was 3.5 times higher in patients who underwent sterilization before the age of 28 years [7]. Patterson et al. [8] also found that African‐American women had an increased risk of pregnancy after sterilization. However, recurrent ectopic pregnancy following a bilateral salpingectomy is extremely rare, with only a few cases reported in the literature [1].

Given the rarity of the condition, we present a unique case of recurrent ectopic pregnancies after bilateral partial salpingectomy. This case serves to highlight the importance of ongoing clinical vigilance despite the rarity of the condition and offers recommendations for the management of ectopic pregnancies in patients with undesired fertility.

## 2. Case Presentation

This is a case of a 28‐year‐old G5P1031 African‐American patient with a dichorionic diamniotic twin pregnancy who delivered at 33 weeks + 4 days via primary low transverse cesarean section after preterm prelabor rupture of membranes (PPROMs) in the setting of vasa previa. Both babies were delivered without any complications and were stable postpartum. After extensive antenatal counseling, the patient opted for bilateral partial salpingectomy. This was performed successfully intraoperatively utilizing the Parkland method. The length of the fallopian tubes removed was 1.5 cm on the left and 0.6 cm on the right. After meeting all postoperative milestones, the patient was discharged home on postoperative day #2 in stable condition. At her 4–5‐week postpartum visit, she reported feeling well overall and resuming menses and intercourse.

Ten months after discharge, now 29 years old G5P1133, the patient presented to the emergency department (ED) with 5 days of progressive abdominal pain that was not relieved by over‐the‐counter pain medication. Her last menstrual period was 5 weeks prior, with regular 28‐day cycles. On presentation to the ED, she denied fevers, chills, lightheadedness, and other complaints outside of the abdominal pain. Her vitals were stable except for a blood pressure of 156/100. On examination, she was alert and in no significant distress. Her abdomen was soft, nondistended, with mild suprapubic tenderness, but no rebound or guarding. Her serum HCG was 194 mIU/mL (Table 1). A transvaginal ultrasound (TVUS) revealed a 2.8 cm × 1.8 cm × 3.7 cm right adnexal mass adjacent to the ovary and small to moderate complex free fluid in the pelvic cavity, consistent with a ruptured ectopic pregnancy (Figure 1). Given the concern for a ruptured ectopic pregnancy, the decision was made to proceed with diagnostic laparoscopy. Intraoperatively, a right ruptured tubal ectopic pregnancy with hemoperitoneum was noted (Figure 2). The right fallopian tube was removed using the LigaSure device, resulting in a hemostatic stump (Figure 2). The estimated blood loss was 50 mL. She was discharged home approximately 4 h later without complications. The patient was seen 5 weeks postoperatively in the clinic and was able to meet all postoperative milestones without any complications. Histopathology confirmed the diagnosis of right ectopic pregnancy as a dilated fallopian tube containing decidualized tissue, and focal immature chorionic villi were identified.

Figure 1Ultrasonographic findings during the patient’s first ectopic pregnancy. (A) Presence of a right adnexal mass adjacent to the ovary, measuring 2.8 cm × 1.8 cm × 3.7 cm, which represents an ectopic pregnancy. Small‐to‐moderate complex fluid in the pelvis, representing the presence of hemoperitoneum, which is worrisome for rupture. Measurements were taken using the measurement tool on the PACS imaging platform via Epic. (B) Doppler flow to the adnexal mass.

**Figure:**
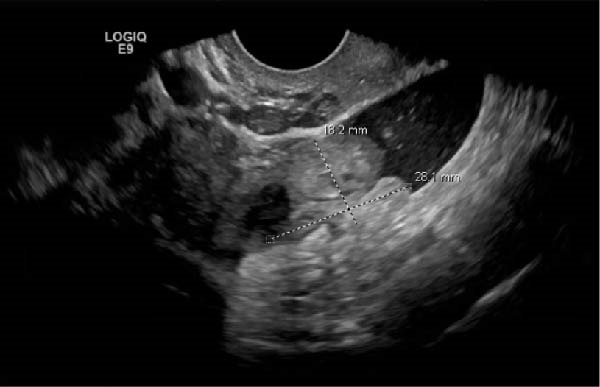
(A)

**Figure:**
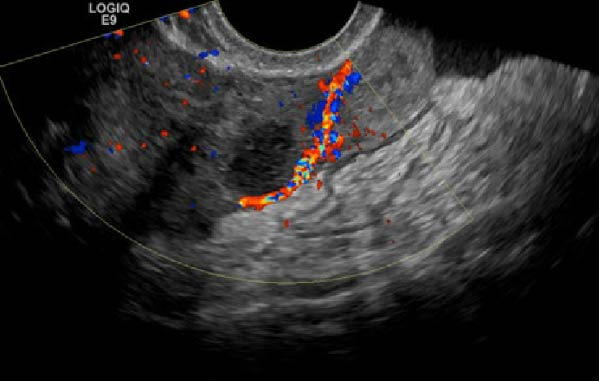
(B)

Figure 2Intraoperative and pathology findings during the first ectopic pregnancy. (A) Presence of hemoperitoneum with adhesions between the uterus and the anterior abdominal wall. (B) Presence of a right tubal ectopic pregnancy. (C) The right part of the pelvis, showing the hemostatic stump after excision of the ectopic pregnancy. (D) The left adnexa at the time, which is remarkable for a congested left infundibulopelvic ligament with evidence of ovarian vein varicosity, with otherwise normally appearing left ovary. (E, F) Histopathology at 2x power field (E) and 4x power field (F). The right fallopian tube contains decidualized tissue and immature chorionic villi.

**Figure:**
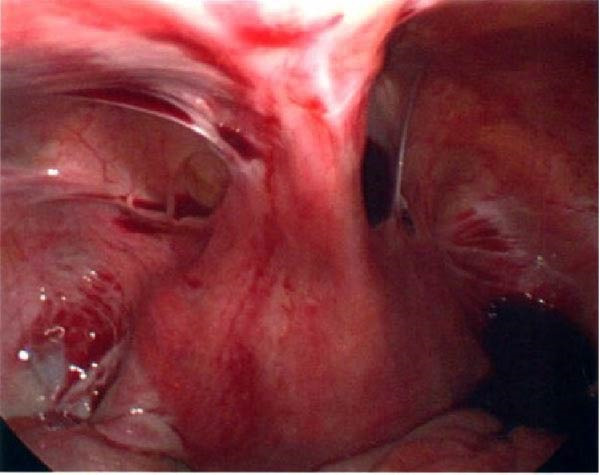
(A)

**Figure:**
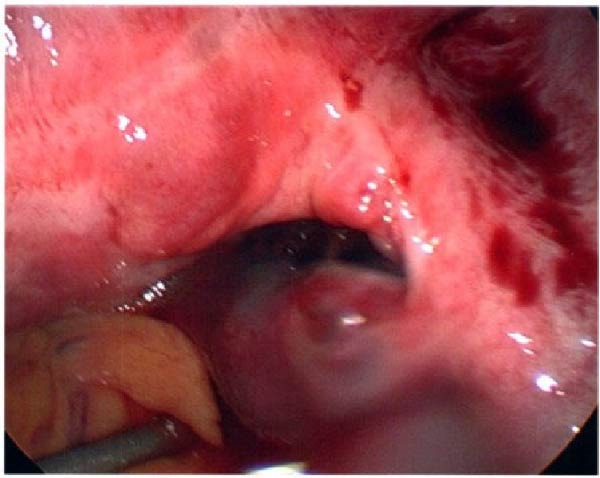
(B)

**Figure:**
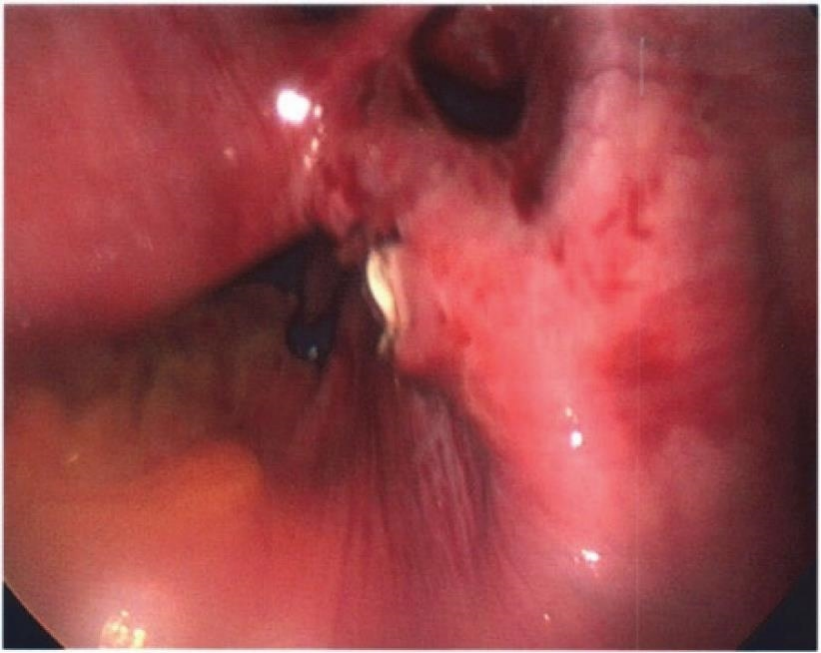
(C)

**Figure:**
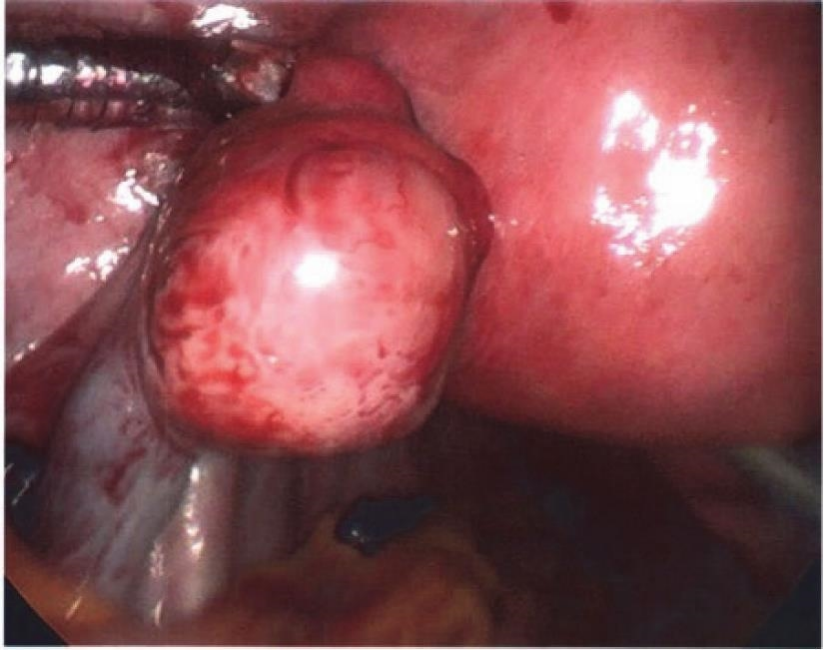
(D)

**Figure:**
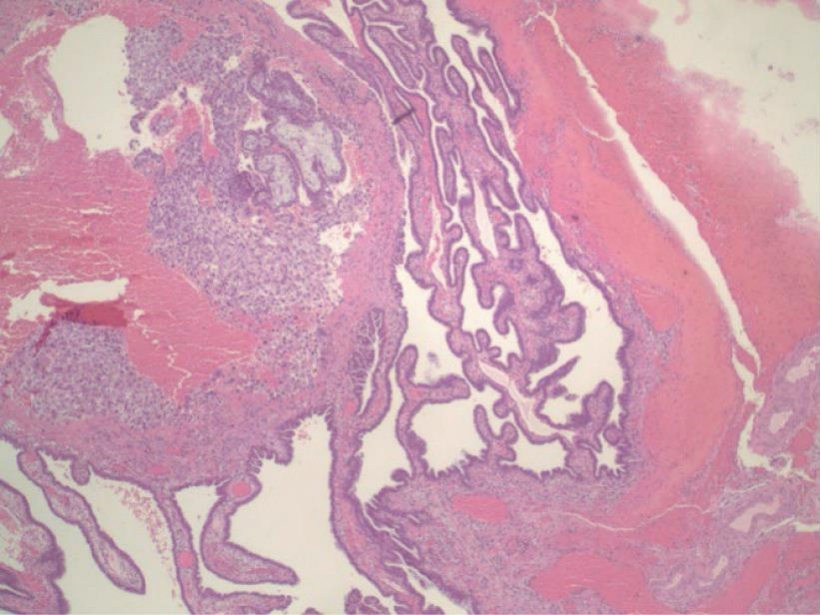
(E)

**Figure:**
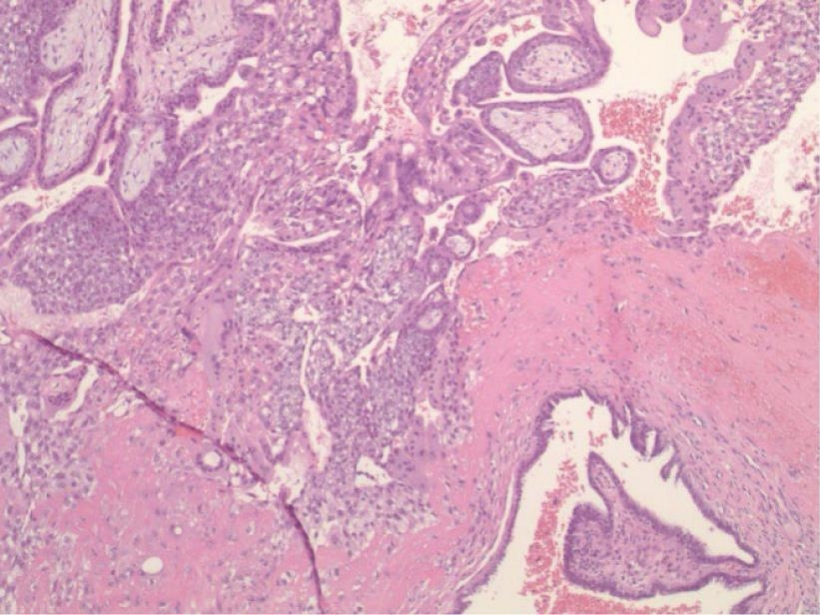
(F)

**Table 1:** Lab findings during the first and second ectopic pregnancy workup.

Ectopic pregnancy #	Lab value	Patient	Normal range
1	β‐hCG (mIU/mL)	194	Nonpregnant patient: <5 Pregnant patient: >25
	Hemoglobin (g/dL)	13.1	12.0–15.5
2	β‐hCG (mIU/mL)	1300	Nonpregnant patient: <5 Pregnant patient: >25
	Hemoglobin (g/dL)	11.2	12.0–15.5

*Note:* Laboratory findings revealed abnormal serum β‐hCG and hemoglobin concentrations during the diagnostic evaluation of the patient’s first and second ectopic pregnancies in the emergency department. Reference ranges for normal values are provided.

**Figure 5:**
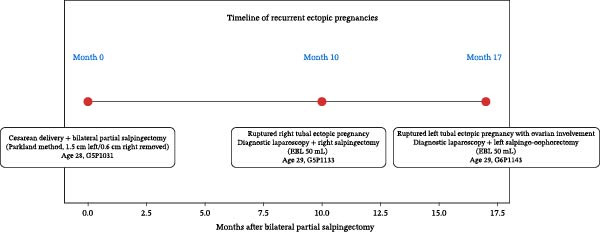
Timeline of recurrent ectopic pregnancies following the initial bilateral partial salpingectomy. Timeline summary of the recurrent ectopic pregnancies starting at month 0 (initial bilateral partial salpingectomy after cesarean delivery) until month 17 (left tubal ectopic pregnancy).

Seventeen months after the initial salpingectomy, now 29 years old G6P1143, the patient presented to the ED with 5 days of worsening left lower quadrant pain and vaginal bleeding. The patient reported that her last menstrual period was 5 weeks prior. Per patient report, the pain gradually worsened and was noted to be similar in nature to the last ectopic pregnancy. She denied nausea, vomiting, vaginal discharge, dizziness, and syncopal episodes. Her vitals were stable except for a blood pressure of 100/74. On examination, she was alert and in no acute distress. Her abdomen was significantly tender to palpation with focal peritoneal signs and guarding in the left lower quadrant. Labs were significant for a serum HCG total of 1300 mIU/mL and mild anemia with hemoglobin of 11.2 g/dL (Table 1). A TVUS revealed a hypoechoic left adnexal mass measuring 4.3 cm × 3.6 cm × 3.0 cm adjacent to the left ovary and complex free fluid in the cul‐de‐sac, consistent with a ruptured ectopic pregnancy (Figure 3). She underwent a second diagnostic laparoscopy. Intraoperatively, a left ruptured tubal ectopic pregnancy with involvement of the left ovary and hemoperitoneum was noted (Figure 4). Left salpingo‐oophorectomy was performed with complete removal of the ectopic pregnancy (Figure 4). Estimated blood loss was 50 mL. She was discharged home 5 h later in stable condition. Histopathology confirmed the diagnosis of left ectopic pregnancy, as a dilated fallopian tube containing blood, decidualized tissue, and immature chorionic villi was identified. The patient did not attend a postoperative clinic visit, but a normal postoperative recovery was confirmed via telephone 2 months postoperatively. The timeline of the recurrent ectopic pregnancies is outlined in Figure 5.

Figure 3Ultrasonographic findings during the patient’s second ectopic pregnancy. (A) Left adnexal hypoechoic lesion seen measuring 4.3 cm × 3.6 cm × 3.0 cm, adjacent to the left ovary, which represents an ectopic pregnancy. Measurements were taken using the measurement tool on the PACS imaging platform via Epic. (B) Complex‐free fluid is seen in the cul‐de‐sac, representing the presence of hemoperitoneum, which is worrisome for rupture.

**Figure:**
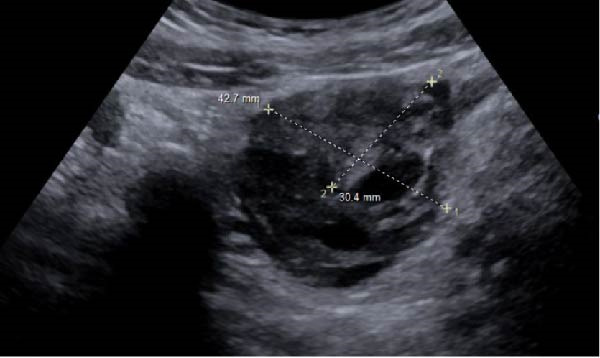
(A)

**Figure:**
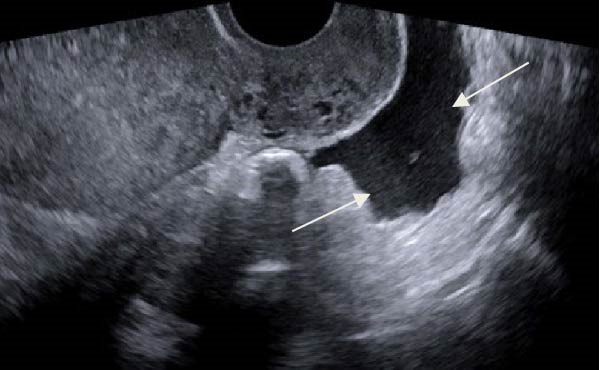
(B)

Figure 4Intraoperative and pathology findings during the second ectopic pregnancy. (A) Presence of hemoperitoneum with a ruptured left ectopic pregnancy involving the left fallopian tube and ovary. (B) Left hemipelvis after complete removal of the ectopic pregnancy via left salpingo‐oophorectomy. (C, D) Histopathology at 2x power field (C) and 4x power field (D). The left fallopian tube contains decidualized tissue and immature chorionic villi.

**Figure:**
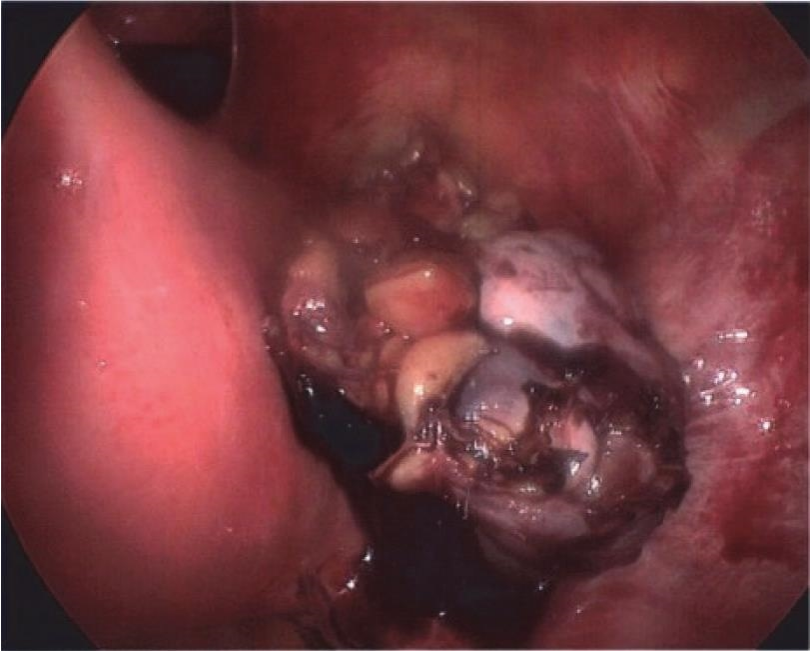
(A)

**Figure:**
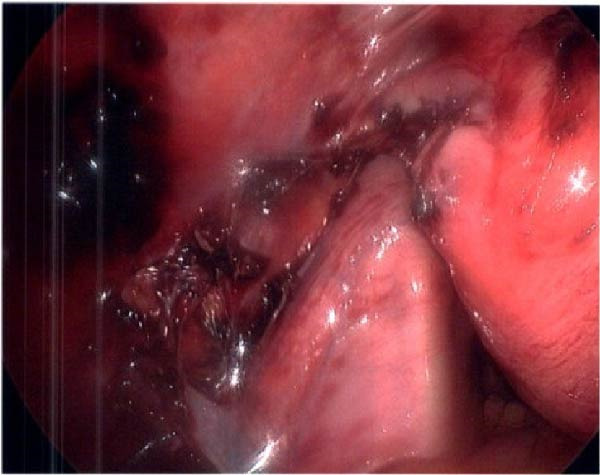
(B)

**Figure:**
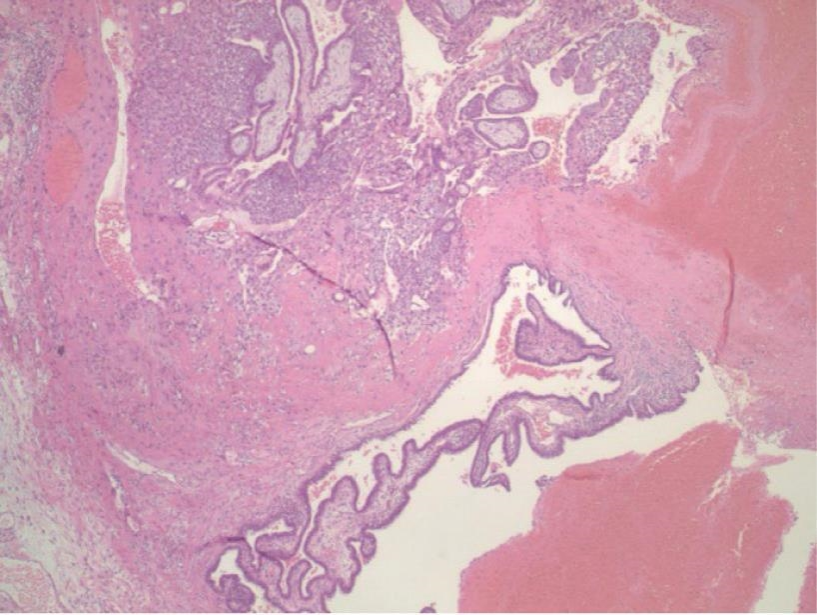
(C)

**Figure:**
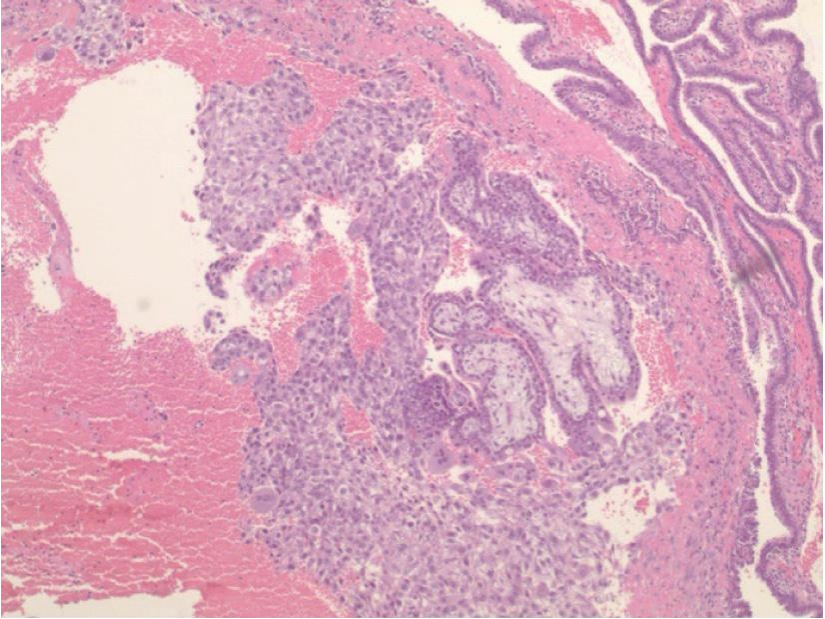
(D)

## 3. Case Discussion

Recurrent ectopic pregnancy following bilateral partial salpingectomy is rare, and the underlying etiology remains uncertain. However, several risk factors have been proposed in the literature. Malacova et al. [7] found that patients who underwent sterilization before the age of 28 years had a 3.5 times higher probability of having an ectopic pregnancy. While the mechanism is not fully understood, one plausible explanation is higher baseline fertility among younger patients [7]. Inflammatory or infectious damage to tubal function and structure from conditions such as sexually transmitted infections and pelvic inflammatory disease may also predispose to ectopic implantation [7]. Moreover, surgical technique impacts risk. Procedures such as laparoscopic electrodestruction and partial salpingectomy carry a higher risk for ectopic pregnancy [7].

Fei et al. [9] noted that physiologic function may persist in tubal remnants; thus, ectopic pregnancy is more likely to occur after a partial salpingectomy than a complete salpingectomy. Mortel and Pacquing‐Songco [1] further hypothesized that tubal remnants longer than 4 cm increase the chance of recanalization. This creates a conduit for fertilization while still preventing intrauterine passage of the ovum, resulting in ectopic implantation [1]. They described an inverse relationship between tubal remnant length and time to development of an ectopic pregnancy [1].

In our case, the patient underwent her initial procedure at the age of 28 years old with no history of pelvic infections. However, her risk was likely elevated due to her younger age, prior pelvic surgery, and African‐American race. Although the Parkland method is the method of choice for partial salpingectomy, it carries a higher risk of ectopic pregnancy compared to complete salpingectomy. Given the average length of fallopian tubes (10–12 cm) and the length removed (1.5 and 0.6 cm), we can extrapolate that more than 4 cm of tubal remnant remained after the partial salpingectomy [10]. At the time of the initial ectopic pregnancy, removal of the contralateral tubal remnant was not performed. The ectopic pregnancy was localized to the right adnexa, and the contralateral tube and ovary appeared grossly normal intraoperatively. Given her prior sterilization, the anticipated risk of future pregnancy was considered low, and additional intervention was deferred to avoid unnecessary operative risk. However, this case highlights that residual tubal segments may retain functional capacity and consideration of contralateral salpingectomy at the time of ectopic pregnancy management may be warranted in selected patients. Furthermore, active tubal remnants, specifically distal fallopian tube fimbriae, are the site of origin for most high‐grade serous ovarian carcinomas [11]. Undergoing a complete salpingectomy has been associated with a 42%–65% reduction in ovarian cancer risk compared with partial salpingectomy or tubal ligation [11].

While the exact cause of recurrent ectopic pregnancy following partial salpingectomy is not fully understood, timely recognition and management are essential to prevent serious morbidity and mortality. In this case, both episodes were successfully managed with diagnostic laparoscopy followed by salpingectomy or salpingo‐oophorectomy, allowing minimal blood loss (50 mL each) and same‐day discharge. Laparoscopy plays a critical role in the management of ectopic pregnancy complications by providing direct visualization of the pathology, precise resection, and hemostasis while minimizing morbidity compared with laparotomy. In similar cases involving tubal remnants, advanced laparoscopic techniques such as resection using endoloops have also been described [12]. Clinicians must remain vigilant, as ectopic pregnancy can still occur after sterilization, particularly within the first 10 years. With younger patients being at higher risk, maintaining a higher clinical suspicion is especially important in this group. Partial salpingectomy, while effective, may leave physiologically active tubal remnants capable of supporting fertilization. This case highlights the importance of considering complete salpingectomy at the time of sterilization when technically feasible. This may reduce the risk of recurrence and improve long‐term outcomes. Comprehensive patient counseling and careful surgical planning are key to enhancing the safety and effectiveness of permanent contraception strategies.

## Funding

No funding was received for this manuscript.

## Consent

We have obtained written informed consent from the patient for publication of this case, including all relevant clinical details and images.

## Conflicts of Interest

The authors declare no conflicts of interest.

## Data Availability

The data that support the findings of this study are available upon request from the corresponding author. The data are not publicly available due to privacy or ethical restrictions.
